# Piloting the objective measurement of eating behaviour at a population scale: a nested study within the Avon Longitudinal Study of Parents and Children

**DOI:** 10.12688/wellcomeopenres.16091.1

**Published:** 2020-08-04

**Authors:** Kaitlin H. Wade, Laura Clifford, Andrew J. Simpkin, Rhona Beynon, Laura Birch, Kate Northstone, Sarah Matthews, George Davey Smith, Julian Hamilton-Shield, Nicholas J. Timpson

**Affiliations:** 1 Medical Research Council (MRC) Integrative Epidemiology Unit, University of Bristol, Bristol, BS8 2BN, UK; 2 Department of Population Health Sciences, Bristol Medical School, University of Bristol, Bristol, BS8 2BN, UK; 3 Department of Paediatric Respiratory Medicine, Bristol Royal Hospital for Children, Bristol, BS2 8BJ, UK; 4 School of Mathematics, Statistics and Applied Mathematics, National University of Ireland, Galway, H91 H3CY, Ireland; 5 NIHR Bristol Biomedical Research Centre Nutrition Theme, University of Bristol, University Hospitals Bristol Education & Research Centre, Bristol, BS1 3NU, UK

**Keywords:** Eating behaviour, obesity, Mandometer, ALSPAC

## Abstract

**Background:** Effective measurement and adaption of eating behaviours, such as eating speed, may improve weight loss and weight over time. We assessed whether the Mandometer, a portable weighing scale connected to a computer that generates a graph of food removal rate from the plate to which it is connected, together with photo-imaging of food, might prove an effective approach to measuring eating behaviours at large scale.

**Methods: **We deployed the Mandometer in the home environment to measure main meals over three days of 95 21-year-old participants of the Avon Longitudinal Study of Parents and Children. We used multi-level models to describe food weight and eating speed and, as exemplar analyses, examined the relationship of eating behaviours with body mass index (BMI), dietary composition (fat content) and genotypic variation (the *FTO* rs9939609 variant). Using this pilot data, we calculated the sample size required to detect differences in food weight and eating speed between groups of an exposure variable.

**Results:** All participants were able to use the Mandometer effectively after brief training. In exemplar analyses, evidence suggested that obese participants consumed more food than those of "normal" weight (i.e., BMI 19 to <25 kg/m ^2^) and that A/A *FTO* homozygotes (an indicator of higher weight) ate at a faster rate compared to T/T homozygotes. There was also some evidence that those with a high-fat diet consumed less food than those with a low-fat diet, but no strong evidence that individuals with medium- or high-fat diets ate at a faster rate.

**Conclusions:** We demonstrated the potential for assessing eating behaviour in a short-term home setting and combining this with information in a research setting. This study may offer the opportunity to design interventions tailored for at-risk eating behaviours, offering advantages over the “one size fits all” approach of current failing obesity interventions.

## Introduction

In many countries, the prevalence of obesity and related complications such as type 2 diabetes continue to increase ^1, 2^. Interventions aimed solely at improving diet and physical activity ignore other potentially modifiable and important factors. For instance, there is evidence that specifically addressing eating behaviours, such as reducing eating speed, may improve weight loss and maintain weight improvement over time ^3, 4^. In 1974, Schachter and Rodin suggested that obese individuals eat at an increased rate, which can disassociate satiety from the amount of food ingested, potentially leading to overeating ^5^. Indeed, an experimental increase in the speed of eating in "normal" weight volunteers (i.e., a body mass index (BMI) ≥19 to <25 kg/m ^2^) caused overeating and delayed the development of satiety, potentially mirroring the pattern of eating in a group of obese patients ^6^. The hypothesis that eating rate is causally related to food intake (and therefore body weight) has been translated into the successful management of adolescent obesity in a randomised controlled trial (RCT) using a device able to record food weight throughout the course of a meal ^4^.

Developed as an intervention to aid food consumption, the Mandometer is a portable weighing scale connected to a small computer that can create a graph representing the rate of food removal from the plate to which it is connected. Removing food from the plate generates a gradual line on the screen visible to the user to monitor speed of consumption (and examine variation in food consumption with changes in eating speed) ^6^. Indeed, using such a device, obese participants successfully modified their eating behaviour by reducing overall speed of eating, whilst maintaining the same level of satiety they had previously experienced with larger portion sizes ^4^. These participants eventually reduced self-determined portion sizes at meals with a sustained response after the therapeutic intervention, which was associated with favourable changes in key satiety hormone responses ^7^. What has not yet been explored is whether the same approaches used to measure eating behaviour can be deployed in a population-based setting for the assessment of normative (and subgroup) trends in eating behaviour.

The association between obesity and eating behaviour has been widely discussed in the literature, with variation in weight playing a role in food consumption and differences in eating behaviour forming a component of obesity predisposition ^8– 12^. Whilst a greater amount of energy (and therefore food) is required to maintain a greater body mass, variation in eating behaviour could, in part, be a cause of the obesity epidemic, with higher food consumption, lower responsiveness to satiety cues and greater responsiveness to external food cues leading to increased weight gain ^8– 12^.

Furthermore, over the last ten years, it has been well documented that various common genome-wide, single nucleotide polymorphisms (SNPs) are associated with both adult and childhood obesity ^13^. As an example, in 2007 it was established that variation in and around the *FTO* gene locus on chromosome 16 was associated with obesity in children and adults ^14, 15^, with studies pointing to the causal variant playing a role in regulation of nearby genes (i.e., *ARID5B*, *IRX3* and *IRX5*) that have downstream effects on thermogenesis to lipid storage, adipocyte size, increased fat stores and body weight gain ^16, 17^. Once this gene had been identified as being associated with obesity, researchers started examining how polymorphic variation impacts on obesity risk ^18^. The A allele of rs9939609 has been linked to a tendency to increased energy dense food consumption ^19^ and increased food intake but decreased satiety responsiveness ^20, 21^. Functional magnetic resonance imaging studies have also demonstrated variation in food cues by *FTO* genotype and hormonal studies have suggested that the same genetic variation may be involved in the regulation of Ghrelin, a key orexigenic hormone, activity ^22, 23^.

Alongside adiposity and related genetic variation, differential dietary composition, macronutrient distribution and patterns are associated with eating behaviours, including consumption quantity and speed ^24, 25^. For example, intake of foods with a high fat content is associated with a higher consumption and eating speed ^26, 27^, possibly explained by the impact of an excess amount of fat on satiation and the impact of increased energy density on energy intake ^28^. Given the complex interplay between adiposity, nutritional content and both internal and external environmental cues to food intake, it remains difficult to disentangle the relationships between eating behaviours and adiposity-related traits.

Together, these studies suggest that unravelling some of the potential mechanisms underlying adiposity, genetic propensity and dietary composition as correlates of eating behaviour may be identifiable and may provide new avenues for therapy. Despite this, methods used to assess these behaviours tend to be labour- and technology-intensive and likely too costly for more extensive population studies. Therefore, this study aimed to assess whether the Mandometer, together with imaging of food to estimate macronutrient content and total calories on the plate, might prove a less intensive and more economical approach to examining eating behaviours.

## Methods

The Avon Longitudinal Study of Parents and Children (ALSPAC) is a large geographically homogeneous prospective birth cohort from the southwest of England established to investigate environmental and genetic characteristics that influence health, development and growth of children and their parents ^29, 30^. Full details of the cohort and study design have been described previously and are available at http://www.alspac.bris.ac.uk. Please note that the study website contains details of all the data that is available through a fully searchable data dictionary and variable search tool ( http://www.bristol.ac.uk/alspac/researchers/our-data/).

Briefly, 14,541 pregnant women residing in the former county of Avon with an estimated delivery date of between the 1 ^st^ of April 1991 and the 31 ^st^ of December 1992 (inclusive) were enrolled to the study. Out of those initially enrolled, 13,998 children who were still alive at 1 year have been followed up to date with measures obtained through regular questionnaires and clinical visits, providing information on a range of behavioural, lifestyle and biological data. Ethical approval for the study was obtained from the ALSPAC Ethics and Law Committee and the Local Research Ethics Committees. Consent for biological samples has been collected in accordance with the Human Tissue Act (2004) and Informed consent for the use of data collected via questionnaires and clinics was obtained from participants following the recommendations of the ALSPAC Ethics and Law Committee at the time. Written informed consent was obtained from mothers at recruitment, from the main carers (usually the mothers) for assessments on the children from ages 7 to 16 years and, from age 16 years onwards, the children gave written informed consent at all assessments.

### Eating behaviour measurement

The Mandometer (Microdiktat, Sweden) was developed at the Section of Applied Neuroendocrinology and Mandometer Clinic, Karolinksa Institutet, Stockholm, Sweden ^4^. The device is a portable weighing scale connected to a small computer that generates a graph representing the rate of food removal from the plate to which it is connected (with weight of food (grams) on the y-axis and time (minutes) on the x-axis). In the therapeutic setting, the user puts a self-determined portion of food on the scale (plate) and the connected computer records and displays to the user, in real-time graphics, the weight loss from the plate as the user eats from time zero (total portion size) to the time they have finished eating.

### Study recruitment and methods

In this feasibility study, we aimed to collect data on eating behaviours (specifically, food weight and eating speed) recorded using the Mandometer in ‘home-based, monitored eating sessions’ within a random sample of the ALSPAC cohort. Recruitment was through a positive response to a randomly selected, mailed-out invitation with information sheet about the study to individuals in the ALSPAC cohort when their mean age was 21 years. Those wishing to take part came to the central, clinic Centre for ALSPAC and went through the study requirements with a research nurse. If happy to take part, they signed a consent form and were then instructed how to use the Mandometer in a baseline data collection clinic at Oakfield House, Bristol. Of the 1,117 invites sent out, 214 accepted and 95 individuals participated in the study.

Over three consecutive days (usually covering one week day and the weekend), participants were asked to go home and eat three separate meals of their choosing (usually dinner) at home recording total weight of meal consumed (grams) and speed of eating in grams/second without the device providing any feedback (so called “blind-meals”). They also took a photograph of each meal using a digital camera with a short description to assess total calorie content and major food types eaten, alongside a questionnaire to provide written detail of the foods consumed.

When three meals had been recorded, the individual under study contacted the research nurse who arranged for a courier to pick up all the equipment. Mandometer data was downloaded on to Mandobase, the central repository for Mandometer data in Sweden. Digital photographs were uploaded on to a central computer prior to analysis. Using the photos and information provided by the participant for each meal, an experienced dietician identified the foods present and visually estimated the weight of each food type before entering data in Dietplan 6 (Forestfield Software Ltd, United Kingdom) ^31, 32^.

Upon recruitment (i.e., when participants were 21 years of age), participants also attended a clinic where they were weighed on Seca scales (nearest 0.1 kg) and their height was measured using a Harpenden Stadiometer (nearest 0.1 cm) whilst wearing light clothing and socks. The participants’ BMI was calculated as weight (kg) divided by height (m) squared.

### Exposure data

As exemplar analyses typical of that likely to be undertaken with data on eating behaviours, we assessed whether eating behaviour was associated with genetic variation, a contemporary measure of BMI and average dietary composition of the meals consumed.

***Genetic variation.*** As part of the ALSPAC study sample, participants were genotyped using the Illumina HumanHap550 quad genome-wide SNP genotyping platform. Participants were excluded due to having at least one of: incorrectly recorded sex, minimal or excessive heterozygosity, disproportionate levels of individual missingness, evidence of cryptic relatedness or non-European ancestry. SNPs with a minor allele frequency (MAF) of <1% and call rate of <95% were removed and only SNPs that passed an exact test of Hardy-Weinberg equilibrium ( *P*<5x10 ^-7^) were included. For this project and at the time of recall, imputation of genotypes was conducted with MACH 1.0.16 Markov Chain Haplotyping software, using CEPH individuals from phase 2 of the HapMap project as a reference (release #22), where imputation quality was high (>0.9). Variation in the *FTO* gene is known to be associated with BMI and explains the most variance in BMI of any known genetic variant (e.g., 0.34%; *P*=4.8x10 ^-120^ reported by Speliotes *et al.* ^33^), and is proposed to do so through appetite and satiety ^34^. Therefore, we used the rs9939609 SNP in the *FTO* gene locus to assess whether BMI is causally related to eating behaviour.

***Variation in BMI.*** For our analyses, BMI measured at the clinic (i.e., when participants were 21 years of age) was categorized into those who were "normal" weight (≥19 to <25 kg/m ^2^), overweight (≥25 kg/m ^2^ and <30 kg/m ^2^) and obese (≥30 kg/m ^2^) according to the WHO guidelines ^35^.

***Dietary composition.*** Using photographs to assist portion size estimations during the Mandometer exercise, we tested whether food weight and eating speed were associated with the overall fat content of participant meals. Food records were converted into weights and codes, which are linkable to the energy and nutrient content of each food item, using DIDO (Diet in, Data out) software ^36^. This software, originally developed by the MRC Human Nutrition Research Unit in Cambridge, has been widely utilised throughout the ALSPAC cohort study ^37^ and has been shown to provide improved accuracy compared with other dietary assessment systems ^38^. All coding was performed by a trained staff member at the Nutrition Theme of the NIHR Bristol Biomedical Research Centre (BRC), University Hospitals Bristol NHS Foundation Trust (previously called the National Institute for Health Research Biomedical Research Unit in Nutrition, Diet and Lifestyle).

Manually coded data were converted to nutrient intakes using a database derived from McCance and Widdowson’s Composition of Foods ^31, 32, 39– 41^. The coding of all food items was checked against the original dietary records. Data cleaning was conducted, and extreme intake values were re-checked to ensure the quality of the data. Estimated meal weights and actual weighed food records from the Mandometer device were comparable, indicating that the coded estimates provide a reliable surrogate measure of dietary intake.

Total energy content (in kilocalories) and macronutrient breakdown (in fat (g), protein (g) and carbohydrate (g)) were calculated for each meal using nutrient data for each individual food item consumed. Macronutrient intakes as a percentage of total energy intake per meal were calculated using standardised energy densities ^32^. These nutrient intake values were then adjusted using the actual meal weight recorded by the Mandometer, to obtain more accurate estimates of the nutritional content of each meal. Finally, an averaged nutritional breakdown of each individual’s recorded meals was calculated.

We partitioned the meals into tertiles according to their overall fat content based loosely on dietary reference intakes ^42^(i.e., low (<30%), medium (≥30% and <35%) and high (>35%) fat), and assessed whether overall fat content was related to their eating behaviour.

### Statistical analysis of Mandometer data

**Figure 1.**
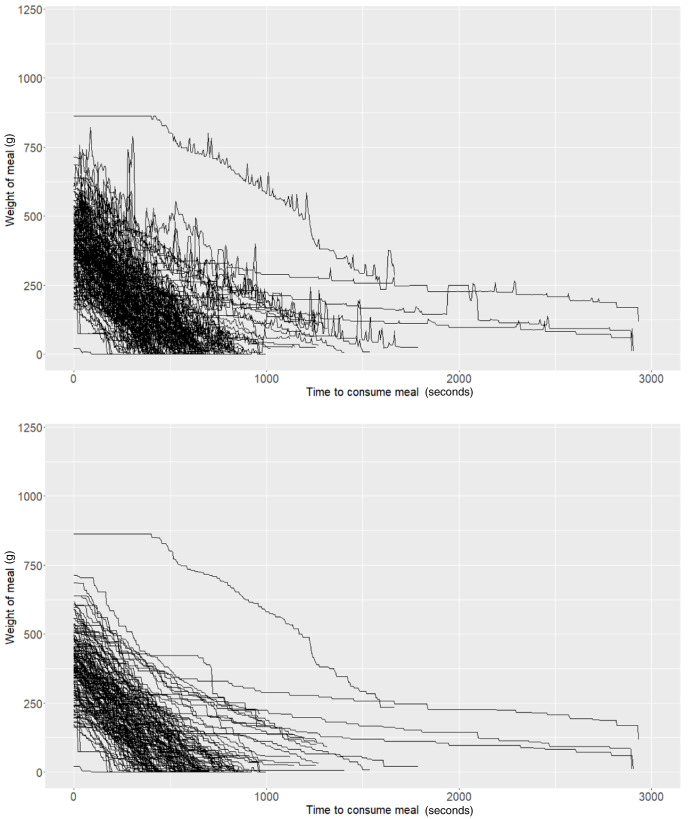
Raw (top panel) and smoothed (bottom panel) Mandometer data (N=50).

As described above, the Mandometer output for each meal can be illustrated as time series data representing the rate of food removal from the plate to which it is connected. In our experiment, we observed “spikes” in the data ( Figure 1) and hypothesised that these could be caused by potential user interactions with the Mandometer (e.g., users placing cutlery on the plate after taking a bite). Attempting to alleviate these potential artefacts, we performed basic smoothing of the data. Here, we only allow weight to decrease. If an increase in weight was detected at a particular snapshot, then the weight was set as the previous snapshot (i.e., we forced the weight to remain constant during these events) ( Figure 1).

Multi-level models were used to account for the hierarchical structure of the data (i.e., repeated measurements of food weight taken within meals and several meals measured within participants). This three-level arrangement was controlled for by allowing one random intercept term for meals and another for participants. These random effects partitioned the variance into (i) between participant, (ii) between meal and (iii) within meal estimates, which account for the non-independence of food weight within meals and within participants. For a given exposure (for example, the *FTO* rs9939609 genotype), the model for food weight is represented by the following equation.

$$\begin{array}{l} mass_{ijk}\;\text{=}\;\beta_{\text{0}}\;+\;u_{0i}\;+\;v_{0ij}\;+\;(\beta_{\text{1}}\;+\;u_{1i}\;+\;v_{1ij})time_{ijk}\;+\;\beta_{\text{2}}1_{A/T}\;+\;\beta_{\text{3}}1_{A/A}\\ \;\;\;\;\;\;\;\;\;+\;\beta_{\text{4}}time_{ijk}\;\times \;1_{A/T}time_{ijk}\;+\;\beta_{\text{5}}time_{ijk}\;\times \;1_{A/A}+\varepsilon_{ijk} \end{array}$$

where *i* = 1, ... , *n* indexes the participant, *j* = 1, ... *n _i_* indexes the meal, *k* = 1, ... , *n _j_* are the repeated measurements of food weight within each meal. *u*_0 *i*_ is the random intercept for each participant *i*, which allows participants to have different average starting meal weights from the overall sample intercept ( *β*_0_). *v*_0 *ij*_ is a random intercept for meals within participants, which allows meals to have different weights. The random slope terms *u*_1 *i*_ and *v*_0 *i*_ allow different eating speeds for participant *i* and meal *j* within participant *i*, respectively.

1 _*A/T*_ is an indicator variable, which takes the value 1 if participant *i* has an A/T genotype and 0 otherwise and, similarly, 1 _*A/A*_ is an indicator for participant *i* having a A/A genotype (here, T/T is the reference group). *β*_1_ gives the average change in meal weight for each unit of *time _ijk_* (seconds) or the rate at which food is consumed; *β*_2_ gives the mean difference in average meal weight between T/T and A/T participants; *β*_3_ gives the mean difference in average meal weight between T/T and A/A participants; *β*_4_ is the mean difference in average eating rate between T/T and A/T participants; and *β*_5_ is the mean difference in average eating rate between T/T and A/A participants. *ε*_*ijk*_ captures the within meal variability, which has a constant variance $\sigma_{\varepsilon }^{2}$ . A value of zero shows that each meal is eaten in a perfectly linear fashion and that there is no error about this line.

### Sensitivity analysis

We carried out a simple multi-level model comprising only random intercepts for individuals and meals to try and realise gross effects. This assumes that participants in each category eat at the same speed, which allows all of the variation in eating speed to be attributed to the individual categories; thereby, making it easier to detect differences between them that are otherwise difficult to determine with low sample sizes.

### Power

Using this pilot data, we were able to carry out a sample size calculation to find the number of individuals required to adequately power (80%) an analysis of eating speed and food weight across BMI, the *FTO* genotype or nutritional categories. Given the lack of previous results in this area, the effect size to detect was set by convention to 0.5 standard deviations (SDs) of food weight and eating speed. The SD of food weight was straightforward to calculate from the observed data. Eating speed was calculated using the best linear unbiased predictor (BLUP) for each meal, and the SD was calculated across these eating speeds.

Using multiple meals per individual leads to clustered data (i.e., each new meal for the same individual is not entirely independent). We present sample sizes required if each individual were to eat two, three or four meals each. The total number of meals needed can then be calculated by multiplying the original sample size by the design effect. This is calculated as 1+(k-1)×ICC, where k is the cluster size (i.e., two, three or four) and the ICC (intra-class correlation) is the proportion of variation in the outcome that is accounted for by the between-meal variation. The ICC is calculated from the multi-level models as the ratio of the between meal variation (i.e., the random intercept and slope variance for *v*_0 *ij*_ and *v*_1 *ij*_) to the total variation.

## Results

Of the 95 people who participated in the Mandometer exercise, there were 89, 60 and 81 participants who had measured BMI, genotype and nutritional information available, respectively, with 54 having all three alongside descriptive characteristics ( Table 1). Approximately one-fifth of these participants were male (25.93%), with a mean age of 21 years (SD = 0.66) at the time of this study. On average, individuals had a BMI of 24.10 kg/m ^2^ (SD = 4.27). The *FTO* genotype was observed at an average MAF of 0.44, where 15% of the participants had the A/A genotype. Across this subsample of participants who undertook the Mandometer exercise and had BMI, genetic and nutritional information, the average starting food weight for each meal was 355.74 g (SD = 155.92) and average time taken to eat meals was 544.76 seconds (SD = 448.37) across a total of 226 meals ( Table 1).

**Table 1.** Sample characteristics of individuals with measures of body mass index, the *FTO* genotype and nutritional information available (N=54).

Variable	N	Mean (SD) or percentage
Sex (% male)	54	25.93
Age (years) of clinic	54	21.02 (0.66)
Average time taken (seconds) to eat meal	54	544.76 (448.37)
Average starting food weight (g) for meal	54	355.74 (155.92)
BMI (kg/m ^2^)	54	24.10 (4.27)
BMI categories	54	
Normal	36	66.67
Overweight	14	25.93
Obese	<5	7.41
*FTO* rs9939609 genotype	54	
T/T	14	25.93
A/T	32	59.26
A/A	8	14.81
MAF of *FTO* rs9939609	54	0.44
Fat Content	54	
Low (<30%)	14	25.93
Medium (≥ 30% <35%)	15	27.78
High (>35%)	25	46.30

BMI = body mass index; MAF = minor allele frequency; SD = standard deviation.

All participants were able to use the Mandometer effectively at home after brief training. Food composition analysis was inconsistent due to blurred photographic images, but descriptions of these meals provided helpful information.

### BMI groups

**Figure 2.**
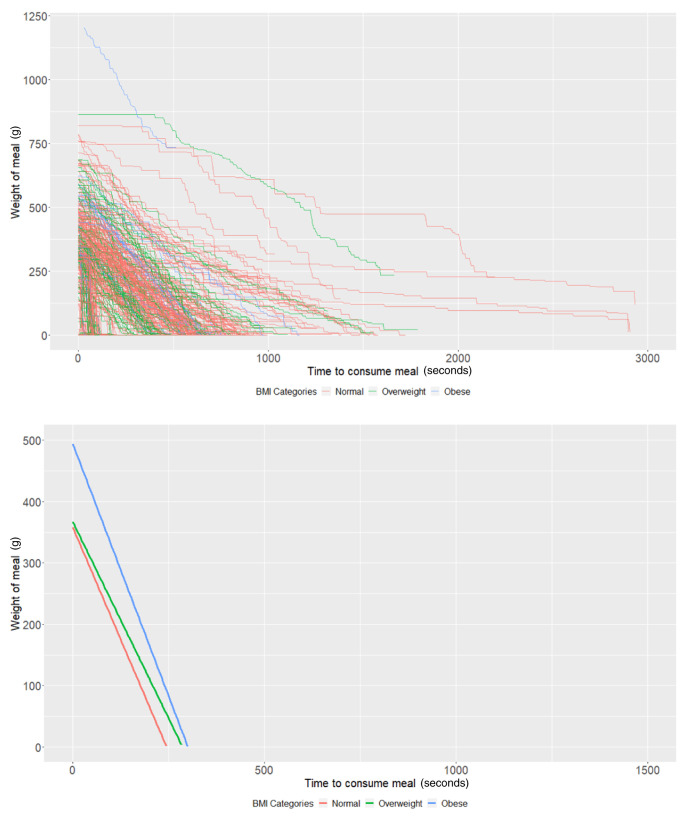
Smoothed output from the Mandometer device stratified by body mass index (BMI, top panel), and relationships between meal weight and eating behaviour stratified by BMI (bottom panel) (N=89).

On average, participants with a "normal" BMI at age 21 years (intercept of our multi-level models) started with 358.09g of food (95% CI: 335.03, 381.14) and ate at a rate (baseline slope of our multi-level models) of 1.46g/second (95% CI: 1.22, 1.70). Obese participants started with 136.17g larger meals on average (95% CI: 52.19, 220.15; *P*=0.001) compared to participants with a "normal" weight ( Table 2). There was no strong evidence ( *P*=0.70) that overweight participants had larger meals than participants with a "normal" BMI. In addition, there was no strong evidence that overweight ( *P*=0.45) and obese ( *P*=0.68) participants ate at a faster rate than participants with a "normal" BMI ( Table 2 and Figure 2). 

**Table 2.** Results from the multi-level model of Mandometer eating behaviour across three meals stratified by body mass index (BMI) (N=89).

Category	Estimate (95% CI) ^1^	*P*-value
Average meal weight (normal BMI; g)	358.09 (335.03, 381.14)	1.49x10 ^-203^
Average difference in meal weight (overweight vs. normal BMI; g)	9.02 (-36.41, 54.46)	0.70
Average difference in meal weight (obese vs. normal BMI; g)	136.17 (52.19, 220.15)	0.001
Average eating speed (normal BMI; g/s)	1.46 (1.22, 1.70)	6.64x10 ^-33^
Average difference in eating speed (overweight vs. normal BMI; g/s)	-0.18 (-0.65, 0.29)	0.45
Average difference in eating speed (obese vs. normal BMI; g/s)	0.18 (-0.69, 1.06)	0.68

^1^Estimates represent the average difference in meal weight (g) or eating speed (g/s) in participants with a “normal” BMI, in overweight participants vs. participants with a “normal” BMI or in obese participants vs. participants with a “normal” BMI (where indicated in the “Category” column).

### *FTO* rs9939609 genotypes

**Figure 3.**
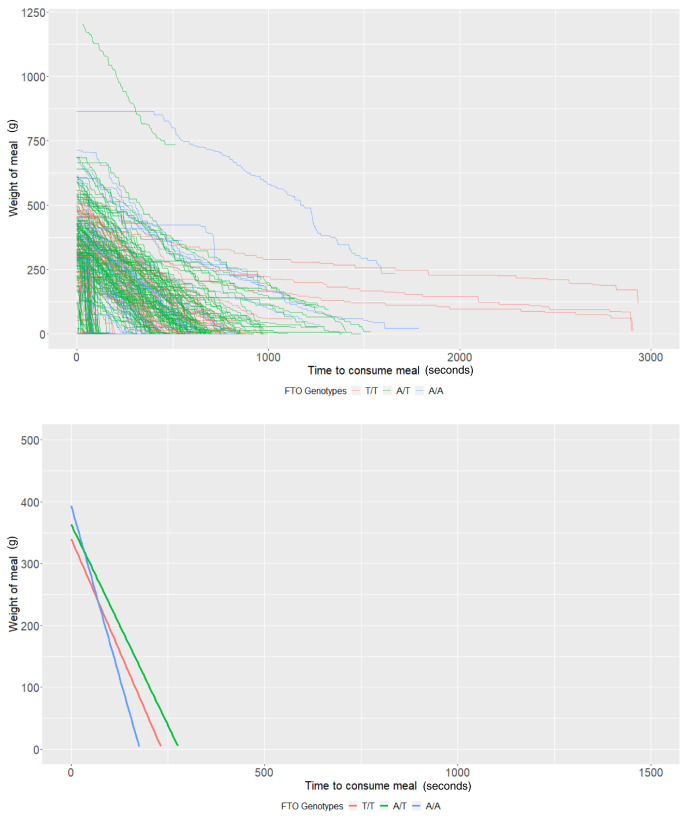
Smoothed output from the Mandometer device stratified by *FTO* genotype (top panel), and relationships between meal weight and eating behaviour stratified by *FTO* genotype (bottom panel) (n=60).

The average meal weight for the T/T genotype (intercept of our multi-level models) was 339.83g (95% CI: 290.36, 389.31) and individuals with this genotype ate at a rate (baseline slope of our multi-level models) of 1.45g/second (95% CI: 0.94, 1.96). There was little evidence that heterozygote individuals (A/T, *P*=0.44) or homozygote individuals for the risk allele (A/A, *P*=0.17) consumed more food. There was some evidence that homozygote individuals ate at a faster rate (0.77g/second faster; 95% CI: -0.01, 1.55; *P*=0.05); however, there was no strong evidence that heterozygote individuals ( *P*=0.63) consumed food at a faster rate ( Table 3 and Figure 3). Due to the relative sample size of genotype groups, estimates are imprecise.

**Table 3.** Results from the multi-level model of Mandometer eating behaviour across three meals stratified by *FTO* genotype (N=60).

Category	Estimate (95% CI) ^1^	*P*-value
Average meal weight (T/T genotype; g)	339.83 (290.36, 389.31)	2.58x10 ^-41^
Average difference in meal weight (A/T vs. T/T genotype; g)	23.36 (-35.62, 82.34)	0.44
Average difference in meal weight (A/A vs. T/T genotype; g)	53.69 (-22.99, 130.36)	0.17
Average eating speed (T/T genotype; g/s)	1.45 (0.94, 1.96)	2.24x10 ^-08^
Average difference in eating speed (A/T vs. T/T genotype; g/s)	-0.15 (-0.75, 0.45)	0.63
Average difference in eating speed (A/A vs. T/T genotype; g/s)	0.77 (-0.01, 1.55)	0.05

^1^Estimates represent the average difference in meal weight (g) or eating speed (g/s) in participants with a homozygous T/T genotype, in participants with a heterozygous A/T genotype vs. participants with a homozygous T/T genotype, or in participants with the homozygous A/A genotype vs. participants with a homozygous T/T genotype (where indicated by the “Category” column).

### Fat content

**Figure 4.**
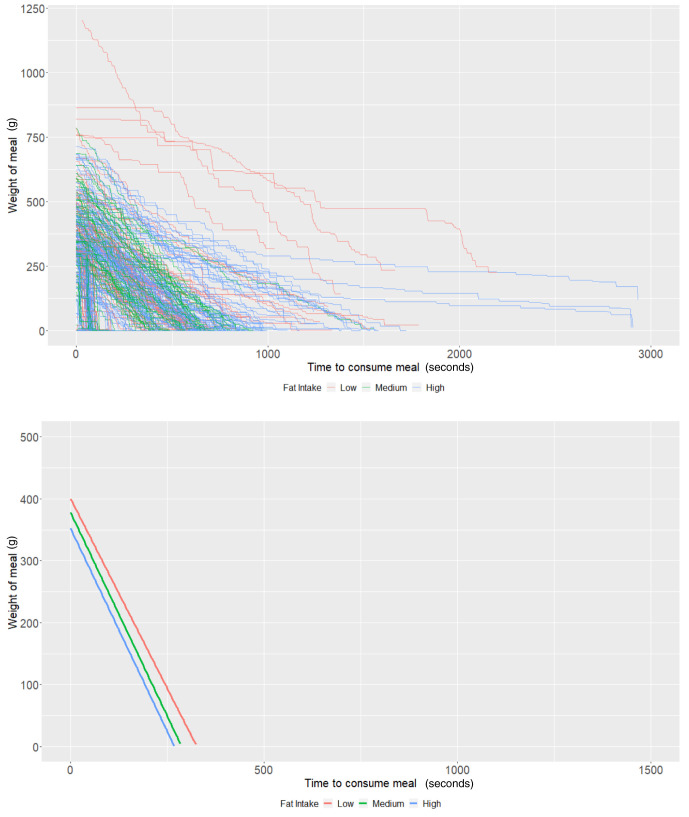
Smoothed output from the Mandometer device stratified by total fat content (top panel), and relationships between meal weight and eating behaviour stratified by total fat content (bottom panel) (N=81).

On average, individuals who consumed a low-fat diet using the Mandometer (i.e., <30% fat across 3 meals) started off (intercept of our multi-level models) with 400.25g of food (95% CI: 356.53, 443.96) and ate at a rate (baseline slope of our multi-level models) of 1.23g/second (95% CI: 0.91, 1.54). There was some evidence that those with a high-fat diet consumed -48.28g less food (95% CI: -101.76, 5.19; *P*=0.08), while there was no strong evidence that those with a medium-fat diet ( *P*=0.49) ate more. Furthermore, there was no strong evidence that individuals with a medium fat diet ( *P*=0.70) or a high-fat diet ( *P*=0.66) consumed food at a faster rate ( Table 4 and Figure 4). As with the other groups, the estimates are imprecise due to the low sample size.

**Table 4.** Results from the multi-level model of Mandometer eating behaviour across three meals stratified by total fat content (N=81).

Category	Estimate (95% CI) ^1^	*P*-value
Average meal weight (low fat; g)	400.25 (356.53, 443.96)	5.19x10 ^-72^
Average difference in meal weight (medium fat vs. low fat; g)	-22.47 (-86.82, 41.88)	0.49
Average difference in meal weight (high fat vs. low fat; g)	-48.28 (-101.76, 5.19)	0.08
Average eating speed (low fat; g/s)	1.23 (0.91, 1.54)	1.54x10 ^-14^
Average difference in eating speed (medium fat vs. low fat; g/s)	0.09 (-0.37, 0.55)	0.70
Average difference in eating speed (high fat vs. low fat; g/s)	0.08 (-0.30, 0.47)	0.66

^1^Estimates represent the average difference in meal weight (g) or eating speed (g/s) in participants with a low fat meal content, in participants with a medium fat vs. low fat meal content, or in participants with a high fat vs. low fat meal content (where indicated by the “Category” column).

### Sensitivity analyses

**Figure 5.**
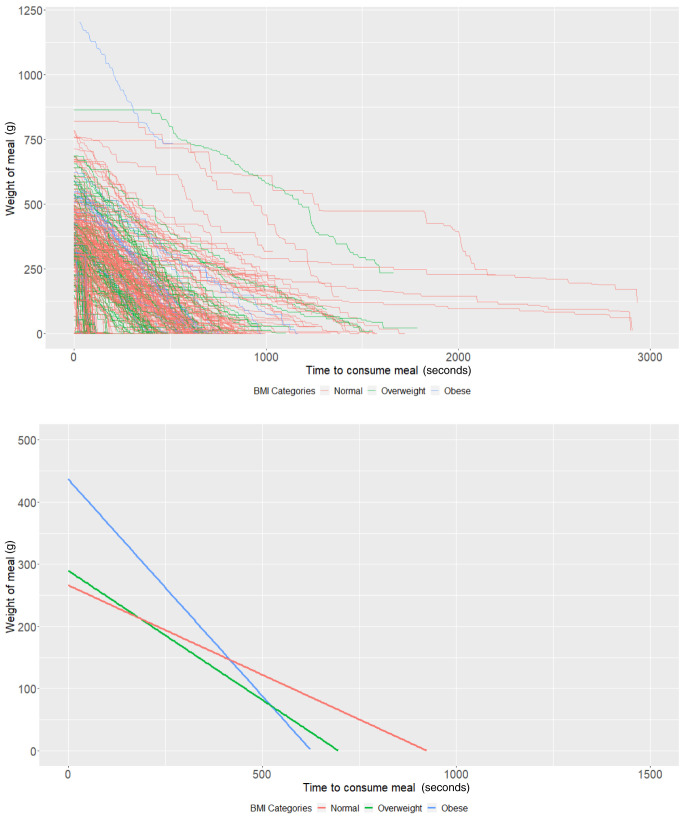
Smoothed output from the Mandometer device stratified by body mass index (BMI, top panel), and relationships between meal weight and eating behaviour (bottom panel) stratified by BMI (using random intercepts only for individuals and meals) (N=89).

Using the random intercept model, the average meal weight for "normal" weight category was estimated to be 266.23g (95% CI: 246.04, 286.43). On average, participants with a "normal" BMI consumed food at a rate of 0.29g/second (95% CI: 0.29, 0.29). In this model, there was evidence that obese individuals had larger meals than individuals with a "normal" BMI (170.67g; 95% CI: 97.11, 244.23; *P*<0.0001). In addition, there was evidence that overweight and obese participants ate at a faster rate than participants with a "normal" BMI ( *P*<0.0001). Overweight participants ate 0.13g/second quicker (95% CI: 0.12, 0.13) than participants with a "normal" BMI, while obese participants ate 0.41g/second faster on average (95% CI: 0.39, 0.42) ( Table 5, Figure 5).

**Table 5.** Results from the multi-level model (using random intercepts for individuals and meals) of Mandometer eating behaviour across three meals stratified by body mass index (BMI) (N=89).

Category	Estimate (95% CI) ^1^	*P*-value
Average meal weight (normal BMI; g)	266.23 (246.04, 286.43)	3.26x10 ^-147^
Average difference in meal weight (overweight vs. normal BMI; g)	23.47 (-16.35, 63.29)	0.25
Average difference in meal weight (obese vs. normal BMI; g)	170.67 (97.11, 244.23)	5.43x10 ^-06^
Average eating speed (normal BMI; g/s)	0.29 (0.29, 0.29)	<1x10 ^-299^
Average difference in eating speed (overweight vs. normal BMI; g/s)	0.13 (0.12, 0.13)	<1x10 ^-299^
Average difference in eating speed (obese vs. normal BMI; g/s)	0.41 (0.39, 0.42)	<1x10 ^-299^

^1^Estimates represent the average difference in meal weight (g) or eating speed (g/s) in participants with a "normal" BMI, in overweight participants vs. participants with a "normal" BMI or in obese participants vs. participants with a "normal" BMI (where indicated in the “Category” column).

**Figure 6.**
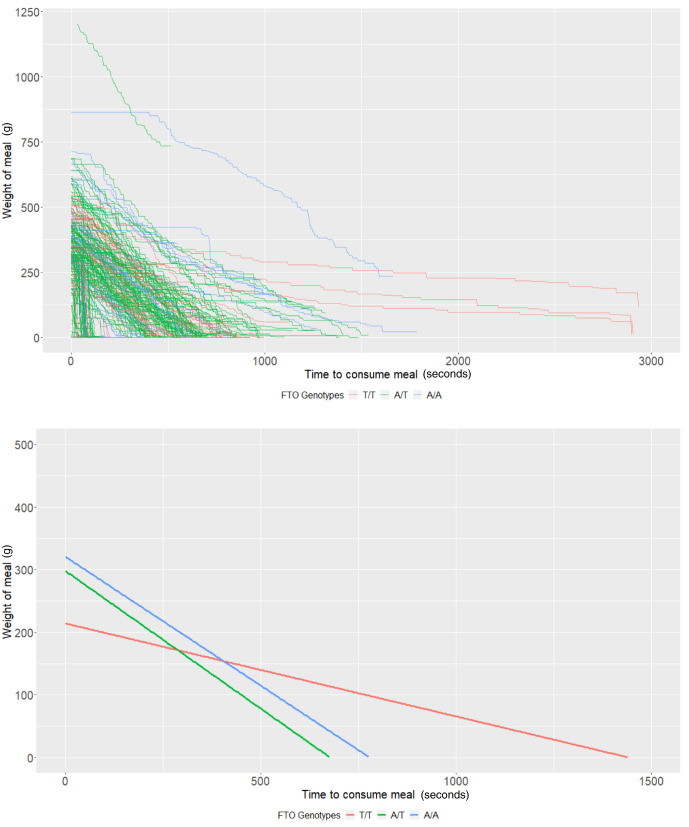
Smoothed output from the Mandometer device stratified by genotype (top panel), and relationships between meal weight and eating behaviour (bottom panel) stratified by genotype (using random intercepts only for individuals and meals) (N=60).

The average meal weight for the T/T genotype was estimated to be 214.14g (95% CI: 176.32, 251.97) and individuals with this genotype consumed food at a rate of 0.15g/second (95% CI: 0.15, 0.15). There was evidence that individuals with the A/T and A/A genotype had larger meals ( *P*=0.0003). Those with the A/T genotype had 83.68g more food (95% CI: 38.57, 128.79) and those with the A/A genotype had 106.39g more food (95% CI: 48.30, 164.48). Furthermore, there was evidence that the A/T and A/A genotypes consumed food at a faster rate ( *P*<0.0001). Those with the A/T genotype ate 0.29g/second faster than those with the T/T genotype (95% CI: 0.29, 0.30), and those with the A/A genotype ate 0.26g/second faster than those with the T/T genotype (95% CI: 0.26, 0.27) ( Table 6, Figure 6).

**Table 6.** Results from the multi-level model (using random intercepts for just individuals and meals) of Mandometer eating behaviour across three meals stratified by genotype (N=60).

Category	Estimate (95% CI) ^1^	*P*-value
Average meal weight (T/T genotype; g)	214.14 (176.32, 251.97)	1.31x10 ^-28^
Average difference in meal weight (A/T vs. T/T genotype; g)	83.68 (38.57, 128.79)	0.0003
Average difference in meal weight (A/T vs. T/T genotype; g)	106.39 (48.30, 164.48)	0.0003
Average eating speed (T/T genotype; g/s)	0.15 (0.15, 0.15)	<1x10 ^-299^
Average difference in eating speed (A/T vs. T/T genotype g/s)	0.29 (0.29, 0.30)	<1x10 ^-299^
Average difference in eating speed (A/A vs. T/T genotype; g/s)	0.26 (0.26, 0.27)	<1x10 ^-299^

^1^Estimates represent the average difference in meal weight (g) or eating speed (g/s) in participants with a homozygous T/T genotype, in participants with a heterozygous A/T genotype vs. participants with a homozygous T/T genotype, or in participants with the homozygous A/A genotype vs. participants with a homozygous T/T genotype (where indicated by the “Category” column).

**Figure 7.**
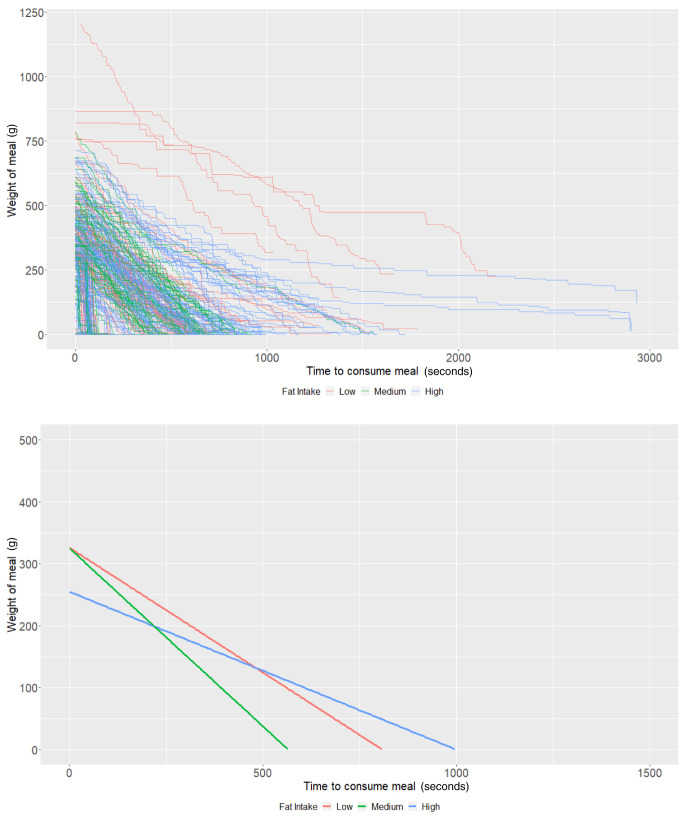
Smoothed output from the Mandometer device stratified by total fat content (top panel), and relationships between meal weight and eating behaviour (bottom panel) stratified by total fat content (using random intercepts only for individuals and meals) (N=81).

Finally, we also observed a similar trend when stratifying by average fat content. The average food weight for individuals with a low-fat diet was 325.94g (95% CI: 290.30, 361.58). Individuals in this group consumed food at a rate of 0.40g/second (95% CI: 0.40, 0.41; *P*<0.0001). In this model, individuals with a medium fat diet had a similar amount of food ( *P*=0.98) but ate at a rate of 0.17g/second faster (95% CI: 0.16, 0.18; *P*<0.0001) than those with a low-fat diet. Those with a high-fat diet ate 70.98g less food (95% CI: 27.41, 114.55; *P*=0.001) and 0.15g/second slower (95% CI: 0.14, 0.15; *P*<0.0001) than those with a low-fat diet ( Table 7, Figure 7).

**Table 7.** Results from the multi-level model (using random intercepts for just individuals and meals) of Mandometer eating behaviour across three meals stratified by total fat content (N=81).

Category	Estimate (95% CI) ^1^	*P*-value
Average meal weight (low fat; g)	325.94 (290.30, 361.58)	7.47x10 ^-72^
Average difference in meal weight (medium fat vs. low fat; g)	-0.83 (-53.29, 51.62)	0.98
Average difference in meal weight (high fat vs. low fat; g)	-70.98 (-114.55, -27.41)	0.001
Average eating speed (low fat; g/s)	0.40 (0.40, 0.41)	<1x10 ^-299^
Average difference in eating speed (medium fat vs. low fat; g/s)	0.17 (0.16, 0.18)	<1x10 ^-299^
Average difference in eating speed (high fat vs. low fat; g/s)	-0.15 (-0.15, -0.14)	<1x10 ^-299^

^1^Estimates represent the average difference in meal weight (g) or eating speed (g/s) in participants with a low fat meal content, in participants with a medium fat vs. low fat meal content, or in participants with a high fat vs. low fat meal content (where indicated by the “Category” column).

### Power

Using the pilot data, the SDs of food weight and eating speed were estimated to be 123g and 0.3g/second, respectively. To achieve 80% power to detect a true difference between groups of 67g in food weight (alpha 0.05), 67 individuals in each group were required. If this difference was additive across BMI categories, then a total sample size of 201 individuals eating one meal each would be required for a three-group exposure variable of interest (i.e., 201 total meals). To adequately power the same analysis for eating speed, 73 individuals were required in each group, or 219 individuals (i.e., 219 total meals) across three, say, BMI groups.

The ICC of meals within individuals was calculated to be 0.97, suggesting that meals were very similar within individuals, and thus multiple meals would not give much extra power and may actually reduce sample size. The design effects for two, three and four meals per person were 1.97, 2.94 and 3.91, respectively. If two meals were recorded for each individual, then 432 total meals (1.97 times the 219 meals estimated for the eating speed analysis above) would be needed, or 216 individuals (432 divided by 2 meals per person - a saving of three people). Similarly, for both three and four meals per person, we would require a sample size of 215 people for both.

## Discussion

In this study, we have shown that it is possible to measure in-depth eating behaviours (i.e., food weight and eating speed) within a healthy population. For future analyses, our calculations suggest that, in order to reliably detect differences in self-selected portion size, we would need 67 individuals in each group (i.e., 201 individuals eating one meal); whereas, to detect differences in eating speed, we would need 73 individuals in each group (i.e., 219 individuals across the three groups). Importantly, these sample sizes reflect the phenotypic precision of the Mandometer and relatively low sample sizes required to achieve resolution in tests by pertinent exposures in this study (i.e., genotype, BMI and meal composition). Other useful details relate to variation in speed of eating across a meal, potentially reflecting satiety responsiveness. It has been demonstrated that those individuals who slow their speed of eating as the meal progresses (so called ‘decelerators’) tend to rate their feeling of fullness higher than linear eaters at the end of a standard meal ^43^. In addition, individuals whose eating speed is naturally linear tend to respond to Mandometer training to eat slower by decreasing food intake.

We used a subsample of the ALSPAC birth cohort to assess the utility of the Mandometer as an instrument to assess eating behaviour at the population level, and whether these eating patterns were associated with BMI, genetic variation associated with BMI and overall nutritional content as exemplar analyses. Using multi-level models, we observed some evidence to suggest that obese participants consumed more food than "normal" weight participants (i.e., BMI ≥19 to <25 kg/m ^2^) and that those with a homozygote *FTO* genetic variant (i.e., an indicator of higher weight) ate at a faster rate. 

We also tested a simpler multi-level model (i.e., one that included random intercepts only for the individual and the meal) as a sensitivity analysis. This simpler model allowed for all the variation in eating speed to be attributed to the individual categories and made differences between the phenotypic groups easier to detect, despite the low sample size. Here, we observed evidence for differences between our phenotypic groups. For example, we observed that overweight and obese individuals consumed more food than those with a "normal" BMI. Furthermore, we also observed that overweight and obese individuals ate at a faster rate when compared to individuals with a "normal" BMI. These trends also replicated across genotypic and nutritional content strata.

In this study, participants each ate several meals meaning that there is a level hierarchy of repeated measures of food weight (i.e., within meals and within participants). To deal with this hierarchy appropriately, we used multi-level models to control for the correlation in meal weight and eating speed within individuals over time and across meals. Findings from sensitivity analyses using only random intercepts in our multi-level models provided some evidence that differences in measured weight categories (i.e., overweight/obese vs. "normal" weight), genotypes (i.e., A/A, A/T vs. T/T) and dietary fat content varied in terms of total food consumption and eating speed. Despite this, not including random slopes in these models are likely not realistic, given they remove inter-individual eating speeds. Therefore, these results should be taken with caution. A further limitation is the assessment of food type and weight on the Mandometer plate, which was assessed by only one experienced dietician, and the limited variability and generalisability in the assessment of only one meal per day, which may not capture full dietary information of the participants. Finally, the use of categorical BMI and fat content groups instead of continuously measured BMI and fat intake reduced power to detect associations between eating speed and these traits. However, this was done to mirror the three-level categories of the *FTO* genetic variant and was a pilot exercise to demonstrate the potential for the future use of the Mandometer as an assessment for eating behaviour.

## Conclusions

This study, together with others (e.g., those that have examined eating behaviours in school children in Sweden ^44^), demonstrated that the Mandometer device has potential in assessing eating behaviour when used in a research setting. It may become an additional tool in nutritional epidemiology for examining phenotype-genotype relationships between pro-adiposity eating behaviours and at-risk polymorphisms. Those studies using fMRI, or other laboratory-based testing such as video analysis of eating behaviour ^45^, offer granular detail but are very costly and time consuming. We have shown that with one clinic visit, which realistically and easily could be transformed into a teaching video, we could examine eating behaviours in the home environment over a period of time. Furthermore, it may offer the opportunity to design interventions that are tailored for genetically determined at-risk eating behaviours offering advantages over the “one size fits all” approach of current obesity interventions that in general achieve only modest improvement in medium to long term adiposity ^46, 47^.

## Data availability

### Underlying data

ALSPAC data are available through a system of managed open access. Data for this project was accessed under the project number B1038. The application steps for ALSPAC data access are highlighted below.

1. Please read the ALSPAC access policy which describes the process of accessing the data in detail, and outlines the costs associated with doing so.
2. You may also find it useful to browse the fully searchable research proposals database, which lists all research projects that have been approved since April 2011.
3. Please submit your research proposal for consideration by the ALSPAC Executive Committee.

You will receive a response within 10 working days to advise you whether your proposal has been approved. If you have any questions about accessing data, please email alspac-data@bristol.ac.uk. The study website also contains details of all the data that is available through a fully searchable data dictionary: http://www.bristol.ac.uk/alspac/researchers/data-access/data-dictionary/.
