# Supplementary material for: Piloting the objective measurement of eating weight and speed at a population scale: a nested study within the Avon Longitudinal Study of Parents and Children
Source: Wellcome Open Res. 2021 Jun 16;5:185. Originally published 2020 Aug 4. [Version 3] doi: 10.12688/wellcomeopenres.16091.3 (PMC8215563; doi:10.12688/wellcomeopenres.16091.3)
Supplement: Peer review history

# Peer review: Reviewer response for version 3

**DOI:** 10.21956/wellcomeopenres.18734.r44503

**Published:** 2021-06-18

**Authors:** Filip Morys

**Affiliations:** 1 Montreal Neurological Institute, McGill University, Montreal, Quebec, Canada

All of my comments have been appropriately addressed and I have no further remarks. Thank you for your work!

Is the work clearly and accurately presented and does it cite the current literature?

Yes

If applicable, is the statistical analysis and its interpretation appropriate?

Partly

Are all the source data underlying the results available to ensure full reproducibility?

Yes

Is the study design appropriate and is the work technically sound?

Yes

Are the conclusions drawn adequately supported by the results?

Yes

Are sufficient details of methods and analysis provided to allow replication by others?

Yes

Reviewer Expertise:

Neurobiological mechanisms of obesity and metabolic syndrome, neuroimaging, eating disorders, eating behaviour

I confirm that I have read this submission and believe that I have an appropriate level of expertise to confirm that it is of an acceptable scientific standard.

# Peer review: Reviewer response for version 2

**DOI:** 10.21956/wellcomeopenres.18248.r43588

**Published:** 2021-05-05

**Authors:** Filip Morys

**Affiliations:** 1 Montreal Neurological Institute, McGill University, Montreal, Quebec, Canada

Wade and colleagues present a pilot study using a relatively large sample size to test the usefulness of a Mandometer in describing eating patterns and relating them to weight status, genotype and dietary fat intake. This is a very carefully conducted and well-designed study which is an excellent introduction to using the Mandometer in future research. The Authors find that meal weight and eating speed differ in groups of individuals with different BMI, genotype and dietary fat intake. More importantly, however, they conduct power analyses to calculate the minimum number of participants to detect between group differences in meal size and eating speed. The Authors are very thorough throughout their analyses and describe the limitations of the study very well. However, some analytical choices were not clearly motivated in the manuscript, some of statistical assumptions were not checked prior to conducting the analyses, and I believe that the Authors could do a slightly better job in describing their results both in terms of being explicit about their sample sizes in each tested group, but also in terms of checking whether their results are accurate, or perhaps driven by outliers or smaller group sizes. I have a small number of mostly minor comments that I hope will help improve this great manuscript.

1. I am a bit puzzled by the general aims of this manuscript. In the abstract the Authors say that the goals are to define interventions tailored for at-risk eating behaviours, yet in the Introduction, they state that the study aims to assess whether the Mandometer is a less intensive and more economical approach to examine eating behaviours. I think a potential for designing interventions, if any, should also be mentioned in the Introduction.
2. Why were the participants divided into groups only based on fat content in their meals? Why not carbohydrates or proteins? This decision needs to be motivated.
3. In the first paragraph of the Results section - 25% is one-fourth, not one-fifth
4. I would like the Authors to discuss how the fact that individuals might have eaten different meals (e.g. breakfast vs. dinner) could affect their results. If, for example, obese participants only ate breakfast using the Mandometer, then their meal weight would presumably be lower. Do the Authors have the information about which meal was eaten? Can this be taken into account in their analyses?
5. It is unclear from the text (but clear from the Tables and Figures) whether the results are presented for the sample of 54 participants or for the full sample for each respective measure.
6. There is only information about distribution of participants in different groups for the sample of 54 participants, but not for other samples. This should be available at least in the supplement.
7. Seeing huge differences in the sample size between obese and lean individuals (but not only) I wonder whether the Authors tested for the assumption of homoscedasticity between the two groups. The variance between groups should be the same, otherwise confidence intervals, parameter estimates and significance cannot be properly calculated in the multilevel models. If the variance is different, this should at least be mentioned as a limitation and I think the Authors should be explicit about their group sizes in the Results section. It is a pilot study after all, so one cannot expect it to be perfect and the outcomes should be judged by the reader, but the reader needs to know what exactly is happening in the described research.
8. I would like to see a scatter plot of starting meal weight and weight status/BMI. From Fig. 2a it seems like the group differences here might be driven by an outlier in the obese group. A similar problem might occur in the genetic analysis. Did the Authors check this?
9. How exactly is the eating speed calculated? Is this measure adjusted for the initial weight of the meal? Would this not make sense?
10. The Authors state that ‘due to the relative sample size of genotype groups, estimates are imprecise’ - why is this different than in the BMI groups? Shouldn’t this statement also be valid for normal weight, overweight and obese groups?

Is the work clearly and accurately presented and does it cite the current literature?

Yes

If applicable, is the statistical analysis and its interpretation appropriate?

Partly

Are all the source data underlying the results available to ensure full reproducibility?

Yes

Is the study design appropriate and is the work technically sound?

Yes

Are the conclusions drawn adequately supported by the results?

Yes

Are sufficient details of methods and analysis provided to allow replication by others?

Yes

Reviewer Expertise:

Neurobiological mechanisms of obesity and metabolic syndrome, neuroimaging, eating disorders, eating behaviour

I confirm that I have read this submission and believe that I have an appropriate level of expertise to confirm that it is of an acceptable scientific standard, however I have significant reservations, as outlined above.

# Response

**Published:** 2021-06-07

**Authors:** Kaitlin Wade

**Affiliations:** University of Bristol, UK

We thank the Reviewer for reading over our manuscript and providing such useful comments. We have responded to each comment below and amended the manuscript accordingly in a manner that we hope is satisfactory.

*Comment 1: I am a bit puzzled by the general aims of this manuscript. In the abstract the Authors say that the goals are to define interventions tailored for at-risk eating behaviours, yet in the Introduction, they state that the study aims to assess whether the Mandometer is a less intensive and more economical approach to examine eating behaviours. I think a potential for designing interventions, if any, should also be mentioned in the Introduction.*

**Response 1: The reviewer is correct, and we apologise for any confusion and inconsistency between the Abstract and Introduction of the manuscript. We have amended by editing the abstract to mirror what is stated in the introduction. As such, the abstract aims now read as follows: “We assessed whether the Mandometer, a portable weighing scale connected to a computer that generates a graph of food removal rate from the plate to which it is connected, together with photo-imaging of food, might prove a less intensive and more economical approach to measuring eating behaviours at large scale”. We have added a small statement about how this device may offer an opportunity to design interventions tailored for at-risk individuals to the last sentence of the introduction.**

*Comment 2: Why were the participants divided into groups only based on fat content in their meals? Why not carbohydrates or proteins? This decision needs to be motivated.*

**Response 2: As this was a pilot study, we explored fat content as an example correlate of eating speed and weight to see whether this could be done for protein and carbohydrates using similar methodology in a larger study. We have now made this clearer in the manuscript.**

*Comment 3: In the first paragraph of the Results section - 25% is one-fourth, not one-fifth.*

**Response 3: Thank you for pointing this out – this has been amended.**

*Comment 4: I would like the Authors to discuss how the fact that individuals might have eaten different meals (e.g., breakfast vs. dinner) could affect their results. If, for example, obese participants only ate breakfast using the Mandometer, then their meal weight would presumably be lower. Do the Authors have the information about which meal was eaten? Can this be taken into account in their analyses?*

**Response 4: The random intercept for each participant included within the multi-level models do allow for differential meal weight within participants; therefore, this is, in part, accounted for in the way that we have modelled the data. However, the reviewer highlights an important point in that it is plausible that individuals in different weight categories may have chosen to systematically record different meals with the Mandometer device. Therefore, the difference in initial meal weight (and potentially eating speed) may be due to the choice of meal recorded by those individuals. It is worth noting, however, that participants were asked to record a “cooked” meal of their choosing (information of which has now been clarified in the manuscript) and this resulted in mainly evening meals being recorded (~90% of meals were eaten after 4pm). Of those who recorded meals that were likely to reflect breakfast or lunchtime meals, only two individuals consistently recorded meals at lunch, both of whom were in the “normal” weight category. Otherwise, all other individuals recorded meals at varying times (again, usually dinner). Although this is unlikely to have impacted our results, it is something that could be of interest in further evaluations in larger samples. This has been added to the discussion as a future consideration.**

*Comment 5: It is unclear from the text (but clear from the Tables and Figures) whether the results are presented for the sample of 54 participants or for the full sample for each respective measure.*

**Response 5: We have added sample sizes where relevant in the results.**

*Comment 6: There is only information about distribution of participants in different groups for the sample of 54 participants, but not for other samples. This should be available at least in the supplement.*

**Response 6: We have now added a table showing the sample characteristics of the pool of 95 participants with non-missing data to the main text and referenced accordingly in the manuscript.**

*Comment 7: Seeing huge differences in the sample size between obese and lean individuals (but not only) I wonder whether the Authors tested for the assumption of homoscedasticity between the two groups. The variance between groups should be the same, otherwise confidence intervals, parameter estimates and significance cannot be properly calculated in the multilevel models. If the variance is different, this should at least be mentioned as a limitation and I think the Authors should be explicit about their group sizes in the Results section. It is a pilot study after all, so one cannot expect it to be perfect and the outcomes should be judged by the reader, but the reader needs to know what exactly is happening in the described research.*

**Response 7: The reviewer has highlighted an important point and having checked this in the current sample, there does not seem to be a substantial difference in the variances between BMI, genotype and fat content groups (box plots provided below). Therefore, whilst unlikely to have a substantial impact in the current study, this is certainly something that needs to be considered in future, larger studies if using similar categorical exposure variables.**

**BMI**

** Genotype**

****

**Fat content**

****

*Comment 8: I would like to see a scatter plot of starting meal weight and weight status/BMI. From Fig. 2a it seems like the group differences here might be driven by an outlier in the obese group. A similar problem might occur in the genetic analysis. Did the Authors check this?*

**Response 8: Given that this was a pilot study demonstrating the utility of the Mandometer device, exploring the impact of a small number of outliers on the results presented was not within the remit of the current analysis. However, this is definitely something that would need to be considered in future evaluations of this relationship in larger studies.**

*Comment 9: How exactly is the eating speed calculated? Is this measure adjusted for the initial weight of the meal? Would this not make sense?*

**Response 9: For each participant, eating speed was calculated as the number of grams consumed by the participant (as measured by the Mandometer device) divided by the number of seconds it took to consume the food (i.e., the slope of the line showing the relationship between seconds on the x-axis and food weight on the y-axis). The equations in the manuscript describe the specific models, where our multi-level models, specifically the random intercepts model, allowed for different mean meal weights and eating speeds between each participant and meal that a participant consumed. The intercept term represents the average initial weight of all meals, with the random intercept allowing this to vary between individuals and between different meals within each individual.**

*Comment 10: The Authors state that ‘due to the relative sample size of genotype groups, estimates are imprecise’ - why is this different than in the BMI groups? Shouldn’t this statement also be valid for normal weight, overweight and obese groups?*

**Response 10: Given that this is a pilot study, it is true that the sample sizes of all groups are small and therefore estimates are more imprecise than what would be achieved with larger sample sizes. We have removed this statement specifically from the section presenting the results of the genotype groups and added this to the discussion as an overall limitation.**

# Peer review: Reviewer response for version 2

**DOI:** 10.21956/wellcomeopenres.18248.r42304

**Published:** 2021-01-25

**Authors:** Falko F. Sniehotta

**Affiliations:** 1 NIHR Policy Research Unit Behavioural Science, Newcastle University, Newcastle, UK

Thanks, nice paper. I don't have any further comments.

Is the work clearly and accurately presented and does it cite the current literature?

Yes

If applicable, is the statistical analysis and its interpretation appropriate?

Yes

Are all the source data underlying the results available to ensure full reproducibility?

Yes

Is the study design appropriate and is the work technically sound?

Yes

Are the conclusions drawn adequately supported by the results?

Yes

Are sufficient details of methods and analysis provided to allow replication by others?

Yes

Reviewer Expertise:

I am a behavioural scientist and have an interest in applied obesity research. I am also interested in measurement reactivity.

I confirm that I have read this submission and believe that I have an appropriate level of expertise to confirm that it is of an acceptable scientific standard.

# Response

**Published:** 2021-05-05

**Authors:** Kaitlin Wade

**Affiliations:** University of Bristol, UK

Thank you!

# Peer review: Reviewer response for version 1

**DOI:** 10.21956/wellcomeopenres.17657.r41015

**Published:** 2020-12-21

**Authors:** Falko F. Sniehotta

**Affiliations:** 1 NIHR Policy Research Unit Behavioural Science, Newcastle University, Newcastle, UK

Congratulations, this is an interesting and well written report. 

 There are just a few odd points I would like to highlight.

1. The title refer to an "objective measurement of eating behaviour" when the Mendometer is mainly an objective measure of consumption quantity and speed. It would be more helpful to make this clear in the title of the paper, not only for future evidence synthesis. 
2. In the context of overweight and obesity the manuscript refers to the term therapy. This term makes all sorts of assumptions about the nature of obesity and the evidence for treatment options which are probably not defensible. Why not referring to intervention? 
3. Surprising that the paper does not refer to reactivity. Monitoring of intake with or without feedback is a common behaviour change technique and the Mandometer has the potential of changing the behaviour it measures. This deserves a mention, here is the latest relevant systematic review https://www.sciencedirect.com/science/article/pii/S0895435619311461 ^1^. 
4. There is a confusion about the nature of the study. It is twice referred to as feasibility study and once as a pilot. What is it? Assuming it is a feasibility study, it would be helpful to clarify what aspect of feasibility was tested and how. In the results, the manuscript reads "All participants were able to use the Mandometer effectively at home after brief training." How exactly was this measured? Also, if it is a feasibility study, this should be reflected in the title. 

Aside from these points, I thoroughly enjoyed reading the paper.

Is the work clearly and accurately presented and does it cite the current literature?

Yes

If applicable, is the statistical analysis and its interpretation appropriate?

Yes

Are all the source data underlying the results available to ensure full reproducibility?

Yes

Is the study design appropriate and is the work technically sound?

Yes

Are the conclusions drawn adequately supported by the results?

Yes

Are sufficient details of methods and analysis provided to allow replication by others?

Yes

Reviewer Expertise:

I am a behavioural scientist and have an interest in applied obesity research. I am also interested in measurement reactivity.

I confirm that I have read this submission and believe that I have an appropriate level of expertise to confirm that it is of an acceptable scientific standard.

## References

[1] : Asking questions changes health-related behavior: an updated systematic review and meta-analysis. *J Clin Epidemiol* .2020;123: 10.1016/j.jclinepi.2020.03.014 59-68 10.1016/j.jclinepi.2020.03.014 32229251PMC7308800

# Response

**Published:** 2021-01-08

**Authors:** Kaitlin Wade

**Affiliations:** University of Bristol, UK

We thank the Reviewer for reading over our manuscript and providing such useful comments and glad that the Reviewer enjoyed our work! We have responded to each comment below and amended the manuscript accordingly in a manner that we hope is satisfactory.

*Comment 1: The title refer to an "objective measurement of eating behaviour" when the Mendometer is mainly an objective measure of consumption quantity and speed. It would be more helpful to make this clear in the title of the paper, not only for future evidence synthesis.*

**Response 1: We totally agree with the reviewer, as we tried to make this clear throughout the text so have mirrored this by changing the title, as suggested by the Reviewer, to “Piloting the objective measurement of eating weight and speed at a population scale: a nested study within the Avon Longitudinal Study of Parents and Children”.**

*Comment 2: In the context of overweight and obesity the manuscript refers to the term therapy. This term makes all sorts of assumptions about the nature of obesity and the evidence for treatment options which are probably not defensible. Why not referring to intervention?*

**Response 2: We agree with the Reviewer and have changed the manuscript such that we discuss the results in the context of possible treatments and interventions, rather than therapy. Specifically, in the introduction, we have changed the word “therapy” in the following sentence such that it now reads as follows: “Together, these studies suggest that unravelling some of the potential mechanisms underlying adiposity, genetic propensity and dietary composition as correlates of eating behaviour may be identifiable and may provide new avenues for future interventions and treatment strategies.”**

*Comment 3: Surprising that the paper does not refer to reactivity. Monitoring of intake with or without feedback is a common behaviour change technique and the Mandometer has the potential of changing the behaviour it measures. This deserves a mention, here is the latest relevant systematic review https://www.sciencedirect.com/science/article/pii/S08954356193114611.*

**Response 3: The point is very well taken. The interactivity of the Mandometer device does indeed have the potential of changing the behaviour it measures and one of the most likely mechanisms by which the Mandometer could be used in interventions/treatment of obesity. We have mentioned this in the conclusive statement of the discussion, specifically: “Furthermore, given the potential of the Mandometer to change eating behaviour via reactivity between the participant and device, it may offer the opportunity to design effective and efficient interventions that are tailored for genetically determined at-risk eating behaviours.”**

*Comment 4: There is a confusion about the nature of the study. It is twice referred to as feasibility study and once as a pilot. What is it? Assuming it is a feasibility study, it would be helpful to clarify what aspect of feasibility was tested and how. In the results, the manuscript reads "All participants were able to use the Mandometer effectively at home after brief training." How exactly was this measured? Also, if it is a feasibility study, this should be reflected in the title.*

**Response 4: We apologise for this uncertainty in the study design – the work we present is indeed more of a pilot study rather than a feasibility study, as the main aim was to undertake a study that could be repeated at larger scale with a similar design. We have removed the “feasibility study” wording and replaced with “pilot study” for clarity. With regards to the inference about participants being able to use the Mandometer, participants were shown how to use the device at a baseline collection clinic by a trained research nurse until the participants felt happy to repeat the process at home on their own, but this was not tested formally – future and larger studies could indeed expand on this and formally test the usage accuracy. We have made this clearer in the “Study recruitment and methods” of the Methods section of the manuscript.**
